# Developing Oligo Probes for Chromosomes Identification in Hemp (*Cannabis sativa* L.)

**DOI:** 10.3390/plants11151900

**Published:** 2022-07-22

**Authors:** Dmitry V. Romanov, Gennady I. Karlov, Mikhail G. Divashuk

**Affiliations:** All-Russia Research Institute of Agricultural Biotechnology, 127550 Moscow, Russia; karlovg@gmail.com (G.I.K.); divashuk@gmail.com (M.G.D.)

**Keywords:** cytogenetic markers, crop, dioecious, oligo-FISH, hybridization, repeat

## Abstract

Hemp (*Cannabis sativa* L., 2n = 20) is a valuable crop that is successfully used as a food, technical and medicinal crop. It is a dioecious plant with an XX\XY sex determination system. Some chromosomes of *C. sativa* have almost the same lengths and centromeric indexes. Cytogenetic markers help to distinguish similar plant chromosomes, including sex chromosomes, which is important for the breeding process. Two repeats (CS-1 and CS-237) were used to develop labeled oligo-probes for rapid and low-cost oligo-FISH. These oligos can be recommended for use as cytological markers to distinguish sex chromosomes (X and Y) and somatic chromosome pairs 3, 6, and 8 by rapid oligo-FISH in a short time.

## 1. Introduction

Hemp (*Cannabis sativa* L.) is an important agricultural plant, that has been cultivated for more than 5000 years [1]. Furthermore, it is one of the oldest plant sources to serve as a food and technical crop [2,3,4,5,6]. *C. sativa* has long been used for medical purposes dating back to ancient times. The plant has medicinal usage in the treatment of burns, pain, glaucoma, nausea, cardiovascular and bronchopulmonary diseases, depression, neuralgia, anemia, and bone fragility, among others [7,8,9,10,11,12,13,14,15].

Plant sex chromosomes are very rare; they have been reported in about 40 species [16,17,18]. *C. sativa* is a dioecious plant with an XX\XY sex determination system [19,20,21,22]; the chromosome number is 2n = 20 [23]. The haploid nuclear genome size for males and females is calculated to be 843 mega-base pairs (Mbp) and 818 Mbp, respectively [24,25]. Sex determination is important for cannabis production and depends on the specific application. For instance, the unfertilized female flowers contain higher levels of cannabinoids and seeded flowers are undesirable for medicinal applications [24]. *C. sativa* can be used for better understanding of the evolution of sex chromosomes [23,26,27]. A high level of polymorphism has been noted in *C. sativa* [28,29]. Furthermore, the chromosomal polymorphism has been detected by FISH on the inter- and intra-cultivar levels of *C. sativa* [23]. The high level of polymorphism determines the importance of *C. sativa* cytological studies for cultivar identification and characterization.

Cytogenetic markers help to distinguish similar plant chromosomes, including sex chromosomes, which is important for the breeding process [19,30]. Cytogenetic markers also help us to associate genomic assemblies with physical chromosomes [31,32]. DNA repeats for FISH analysis usually need to be amplified in a bacterial plasmid, extracted, and then labeled by nick translation [33,34]. In addition, FISH probes can be generated by PCR, but DNA isolation is previously required [35,36,37]. Thus, the procedure for preparing the probes is time consuming. Synthetic oligonucleotides with a fluorescent label can also be used as a probe for FISH [38,39] or ND-FISH analysis [40,41,42,43,44,45,46,47,48]. Such a probe is convenient to use since fluorochrome labeled oligonucleotides can be purchased directly from commercial sources. ND-FISH have a short hybridization time and no denaturation process, saving the structure of chromosomes, reagents and other resources [40,46,47,49]. Based on these advantages, the method is widely used to identify plant chromosomes [39,40,42,47,48]. Moreover, ND-FISH technology can be used to identify crop varieties [50]. ND-FISH is simple and convenient, but less reproducible in a series of experiments [46].

Oligos are being developed for easy application, which allows researchers to obtain results by fast oligo-FISH or ND-FISH in a short time. Oligos have been developed and successfully used for cereals [40,41,42,43,51,52] and other crops [53,54,55]. In this article, we report the development of oligo-labeled probes, based on CS-1 [56] and CS-237 [31] repeats for rapid oligo-FISH. The possibility of their use as cytological markers for *C. sativa* sex and some somatic chromosomes is discussed.

## 2. Results and Discussion

**Figure 1:**
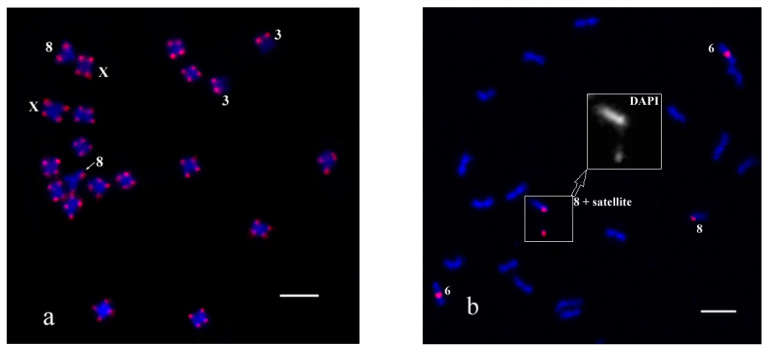
The results of FISH experiments with the PCR labelled probes CS-1 and CS-237 on *C. sativa* chromosomes: (**a**) pink—CS-1 probe; (**b**) pink—CS-237 probe. DAPI layer of chromosome 8 with satellite is enlarged and shown in gray. Distinguished chromosomes are indicated according to Alexandrov et al. [31]. Bar equals 5 μm.

To compare the efficiency between conventional FISH and oligo-FISH we used them in turn. The first experiment was carried out on *C. sativa* chromosomes with PCR labelled probes. As a result, hybridization signals of CS-1 (Figure 1a) and CS-237 (Figure 1b) were detected as described by Divashuk et al. [56] and Alexandrov et al. [31], respectively. Satellites are often separated from the chromosome 8, as shown in Figure 1b.

Labeled oligos were designed and synthesized for the repeats CS-1 and CS-237 by Primer3 software [57] for more simple use of them as cytogenetic markers. For each repeat we designed two different sequences of oligos to increase the chances of successful hybridization. Labeling was carried out by using various fluorophores (FAM, TAMRA, Cy5) (Table 1) for more convenient use in combination with other labeled probes.

**Figure 2:**
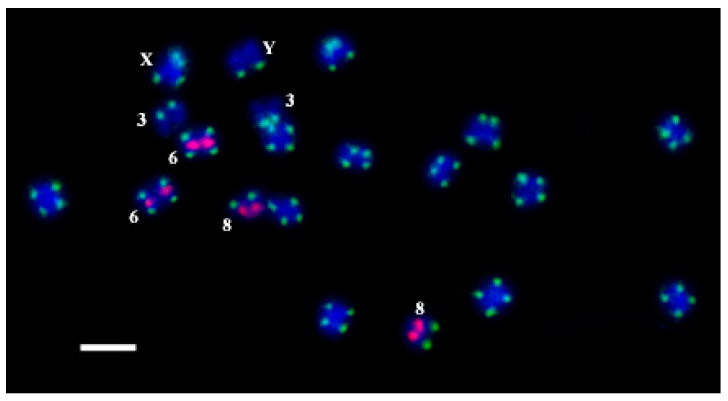
The results of oligo-FISH experiments with the oligo-probes CS-1-FAM (green) and CS-237-TAMRA (pink) on *C. sativa* chromosomes. Distinguished chromosomes are indicated according to Alexandrov et al. [31]. Bar equals 5 μm.

All the oligos were hybridized on *C. sativa* chromosomes by rapid oligo-FISH (Figure 2). Hybridization signals were observed for all the developed oligos. The results of the oligo-FISH probe detection with all the oligos on *C. sativa* chromosomes were similar as for FISH with the PCR labelled probes. There were no non-specific signals interfering with the work with the metaphase plates.

We found that the oligo-probes oligoCS-1-FAM, oligoCS-1-Cy5, and oligoCS-1-TAMRA are located in the subtelomeric region on both arms of all *C. sativa* chromosomes, except for the long arm of chromosome 3, the short arm of chromosome 8, and the long arm of the Y chromosome at the same positions. The oligo-probes oligoCS-237-FAM, oligoCS-237-Cy5, and oligoCS-237-TAMRA are located in the proximal part of the short arm of chromosome 6 and in the distal part of the short arm of chromosome 8 at the same positions. Thus, we can use any oligo with any dye from Table 1 suitable for a particular experiment. The positions of the oligos completely coincide with the positions of the corresponding repeats on *C. sativa* chromosomes [31,56]. We noted a different intensity of signals in different plants of *C. sativa*, which is quite consistent with Razumova et al. [23], where subtelomere hybridization sites disappear or new hybridization sites appear. This interesting phenomenon for the cross-pollinated plant is relatively expected and requires further study.

Some chromosomes of *C. sativa* are difficult to distinguish from each other without the use of cytological markers (chromosomes 5 and 6, X and Y) since they have almost the same lengths and centromeric indexes. Differences in the position of the oligo-probes for CS-1 and CS-237 repeats on *C. sativa* chromosomes make it possible to distinguish chromosomes 5 and 6, X and Y and easily identify chromosomes 3 and 8. Razumova et al. [23] showed different variations of FISH signals for the CS-1 repeat on chromosome 8 and on chromosome X. Nevertheless, the combined use of the oligo-probes for CS-1 and CS-237 repeats can identify these chromosomes. Thus, the probes can be recommended as cytogenetic markers for better identification of *C. sativa* chromosomes 3, 6, 8, X and Y by rapid oligo-FISH.

## 3. Conclusions

In conclusion, the oligonucleotide probes oligoCS-1-FAM, oligoCS-1-Cy5, oligoCS-1-TAMRA, oligoCS-237-FAM, oligoCS-237-Cy5, and oligoCS-237-TAMRA have been developed in the present study. They can be used to conveniently identify sex and somatic chromosomes and their polymorphism in hemp using rapid oligo-FISH.

## 4. Materials and Methods

### 4.1. Plant Material

For the study of mitosis metaphase chromosomes, cv ‘‘Zenitsa’’ (dioecious) seeds of *C. sativa* were used (originated by P.P. Lukyanenko Krasnodar Research and the Development Institute of Agriculture, Krasnodar, Russia). The seeds were harvested in 2020 and then stored at +4 °C.

### 4.2. Chromosome Preparation

*C. sativa* seeds were germinated on moist filter paper in Petri dishes at 24 °C in the dark for 72 h. The seedlings (2–3 cm long) were used to prepare the slides of mitotic metaphase chromosomes as described by Romanov et al. [58].

### 4.3. DNA Probes and Labeling

Young seedlings of *C. sativa* were used for DNA isolation by the CTAB method [59]. Repeat sequences CS-1 and CS-237, previously described by Divashuk et al. [56] and Alexandrov et al. [31], respectively, were used to design primers and oligos by using the Primer3 software v.4.1.0 [57]. DNA labeling was performed by PCR with biotin-16-dUTP according to the manufacturer’s instruction (Boehringer, Germany). The oligos were synthesized and labeled by FAM, TAMRA or Cy5 (Evrogen JSC, Moscow, Russia).

### 4.4. Fluorescence In Situ Hybridization (FISH) and Oligo-FISH

The FISH experiments were performed as described by Karlov et al. [60]. Post-hybridization washing was performed in 50% (*v*/*v*) formamide in 2× SSC for 15 min at 42 °C, while the theoretical washing stringency was about 80%. The chromosomes were counterstained with 1 mg/mL DAPI and mounted in Vectashield (Vector laboratories, Burlingame, CA, USA). Oligo-FISH experiments were performed as FISH, but without the detection step, as described by Kuznetsova et al. [46]. The hybridization procedure for FISH and oligo-FISH lasted for 1 h and 16 h, respectively, at 37 °C.

### 4.5. Microscopy and Image Analysis

Chromosome preparations were viewed by using a THUNDER 3D Tissue microscope with a filter set DFT51111, a fluorescence light source LED3, and a digital camera DFC9000 GTC (Leica Microsystems, Wetzlar, Germany). Multichannel fluorescence recording, image processing for brightness/contrast and color settings were performed using LasX software (Leica Microsystems, Wetzlar, Germany). The karyotype for *C. sativa* metaphase chromosomes was developed by Divashuk et al. [56] and improved by Alexandrov et al. [31]. In this article, chromosome numbering is in accordance with Alexandrov et al. [31]. Chromosomes 3, 5, 6, 8, X, and Y according to Alexandrov et al. [31] correspond to chromosomes 4, 6, 3, 9, X, and Y, respectively, according to Divashuk et al. [56].

## Figures and Tables

**Table 1:** Labeled oligo-probes designed for the repeats CS-1 and CS-237.

Labeled Oligo-Probe	Repeat	Oligonucleotide	Dye
oligoCS-1-FAM	CS-1	5′-TAGTTATCTGTTAAAATCTCAACCTACACA-3′	5`-6-FAM
oligoCS-1-Cy5	CS-1	5′-TAGTTATCTGTTAAAATCTCAACCTACACA-3′	5`-Cy5
oligoCS-1-TAMRA	CS-1	5′-ATCGTTTTAATCGAAATAGTGAAAATCTCA-3′	5`-TAMRA
oligoCS-237-FAM	CS-237	5′-ATGTATTGCTGACACTCATTTGAAATCATC-3′	5`-6-FAM
oligoCS-237-Cy5	CS-237	5′-ATGTATTGCTGACACTCATTTGAAATCATC-3′	5`-Cy5
oligoCS-237-TAMRA	CS-237	5′-TACGTTGGTTGATTGAGGATGTTTGAAA-3′	5`-TAMRA

## Data Availability

Not applicable.
